# Social and Environmental Determinants of Health and Cardio-Kidney-Metabolic Syndrome–Related Mortality

**DOI:** 10.1001/jamanetworkopen.2024.35783

**Published:** 2024-09-26

**Authors:** Pedro Rafael Vieira de Oliveira Salerno, Antoinette Cotton, Yakov E. Elgudin, Salim Virani, Khurram Nasir, Ian Neeland, Sanjay Rajagopalan, Naveed Sattar, Sadeer Al-Kindi, Salil V. Deo

**Affiliations:** 1 Harrington Heart and Vascular Institute, University Hospitals, Cleveland, Ohio; 2 Case School of Medicine, Case Western Reserve University, Cleveland, Ohio; 3 Louis Stokes Cleveland VA Medical Center, Cleveland, Ohio; 4 The Aga Khan University, Karachi, Pakistan; 5 Section of Cardiology, Baylor College of Medicine, Houston, Texas; 6 Texas Heart Institute, Houston, TX; 7 DeBakey Heart and Vascular Center, Houston Methodist Hospital, Houston, Texas; 8 School of Cardiovascular and Metabolic Health, University of Glasgow, Glasgow, United Kingdom; 9 School of Health and Wellbeing, University of Glasgow, Glasgow, United Kingdom

## Abstract

**Importance:**

It is not well understood if and how various social and environmental determinants of health (SEDoH) are associated with mortality rates related to cardio-kidney-metabolic syndrome (CKM) across the US.

**Objective:**

To study the magnitude of the association strength of SEDoH with CKM-related mortality at the county level across the US.

**Design, Setting, and Participants:**

This cross-sectional, retrospective, population-based study used aggregate county-level data from the US Centers for Disease Control and Prevention Wide-Ranging Online Data for Epidemiologic Research (WONDER) data portal from 2010-2019. Data analysis occurred from September 2023 to January 2024.

**Exposures:**

A total of 7 diverse SEDoH were chosen, including median annual household income, percentage of racial and ethnic minority residents per county, fine particulate air pollution (PM_2.5_) concentrations, high-school completion rate, primary health care access, food insecurity, and rurality rate.

**Main Outcomes and Measures:**

The primary outcome was county-level age-adjusted mortality rate (aaMR) attributable to CKM. The association of county-level CKM-related aaMR with the 7 SEDoH was analyzed using geographically weighted models and the model median coefficients for each covariate studied.

**Results:**

Data from 3101 of 3243 counties (95.6%) were analyzed. There was substantial variation in SEDoH between states and counties. The overall pooled median (IQR) aaMR (2010-2019) in the US was 505.5 (441.3-578.9) per 100 000 residents. Most counties in the lower half of the US had rates much higher than the pooled median (eg, Southern US median [IQR] aaMR, 537.3 [466.0-615.9] per 100 000 residents). CKM-related mortality was positively associated with the food insecurity rate (median [IQR] β = 6.78 [2.78-11.56]) and PM_2.5 _concentrations (median [IQR] β = 5.52 [−11.06 to 19.70]), while it was negatively associated with median annual household income (median [IQR] β = −0.002 [−0.003 to −0.001]), rurality (median [IQR] β = −0.32 [−0.67 to 0.02]), high school completion rate (median [IQR] β = −1.89 [−4.54 to 0.10]), racial and ethnic minority rate (median [IQR] β = −0.66 [−1.85 to 0.89]), and primary health care access rate (median [IQR] β = −0.18 [−0.35 to 0.07]).

**Conclusions and Relevance:**

In this cross-sectional study of county-level data across the US, there were substantial geographical differences in the magnitude of the association of SEDoH with CKM-related aaMR. These findings may provide guidance for deciding local health care policy.

## Key Points

**Question:**

Does the magnitude of the association of social and environmental determinants of health (SEDoH) with cardio-kidney-metabolic (CKM) syndrome–related mortality vary across the US?

**Findings:**

In this cross-sectional study of 3101 US counties using geographically weighted models, there was a high level of geographical variation in the magnitude of the association of SEDoH with county-level CKM-related mortality in the US. Among the studied SEDoH, median household income, food insecurity, and high school completion rate were the factors with the most pronounced association with mortality.

**Meaning:**

This cross-sectional study found a differential association of the included SEDoH with CKM-related mortality in the US; these findings should inform local level health care policy decisions.

## Teaser

This cross-sectional study analyzes associations of various social and environmental determinants of health with cardio-kidney-metabolic syndrome mortality rates at the county level across the US.

## Introduction

Cardiovascular disease (CVD) has been an important cause of death in the US for the past many decades.^1^ While CVD remains among the top 5 nonaccidental causes of adult mortality in the US, recent studies suggest that mortality rates related to CVD have reduced in the past decade.^2^ Likely causes that have led to these changes may be better therapeutics, reduced mortality following myocardial infarction, and better outcomes of both percutaneous and surgical intervention. However, apart from these measures, continued initiatives to promote primary preventative measures avoiding or controlling the traditional cardiovascular risk factors, namely, hypertension, diabetes, obesity, and dyslipidemia, have also greatly helped to improve the overall picture of CVD in the US.^3^ However, despite the use of such measures, residual risk for CVD remains. To further reduce incident CVD and improve outcomes, recent research has focused on nontraditional cardiovascular risk factors, namely, social and environmental determinants of health (SEDoH).^4,5^ Multiple prior mechanistic studies have reported associations of SEDoH with the severity of CVD.^4,5,6^ However, unlike traditional cardiovascular risk factors that physicians can attempt to reduce at the patient level, improving the SEDoH requires large, dedicated public health initiatives. These projects are often lengthy, expensive, and require commitment from multiple different organizations for them to be successful.^7^ Given that a recent study reported that social determinants vary greatly across the US, for the appropriate local allocation of resources, it is important to understand which factors are more relevant in which regions.^8^ Many prior studies have investigated the association of various SEDoH with CVD, but have failed to account and appreciate this spatial heterogeneity. Among the available statistical models, geographically weighted regression models can uncover this potential heterogeneity and evaluate the differential association of exposure with outcome based on location.^9,10,11,12^ Therefore, the inference from these models can be used to inform local level public health policymaking.

Given the recent advances in cardiometabolic therapeutics and understanding of the shared causal origins for these phenotypes, the American Heart Association (AHA) recently introduced the concept of the cardio-kidney-metabolic syndrome (CKM).^13^ Unfortunately, while researchers have studied all these conditions separately in much detail, we do not know the current burden of CKM in the US. Realizing these gaps in present knowledge, we obtained county-level all-cause mortality related to underlying CKM in the US and analyzed associations with important SEDoH. In this study, apart from reporting the current burden of CKM mortality, we aimed to explore associations of CMK mortality with SDOH, and whether the magnitude of the associations of different SEDoH factors with CKM mortality varied across the US at the county level. We did so to better understand such associations as a stimulus to better define potentially useful future preventative public health policies.

## Methods

This cross-sectional study was determined to be exempt from review and the requirement of informed consent by the Case Western Reserve University institutional review board. The reporting of this study followed the Strengthening the Reporting of Observational Studies in Epidemiology (STROBE) reporting guideline.

### Selection of County-Level SEDoH

We initially collected information on 52 different exposures from a variety of public sources related to social determinants of health, environmental condition, and health care access (eTable 1 in Supplement 1). Using a combination of expert knowledge, review of prior literature, and current understanding, we chose 7 different SEDoH which encompass the different SEDoH measures that are known to be associated with CVD.^14,15,16,17^ The SEDoH exposures that we finally modeled in our study included (1) racial and ethnic minority rate (defined as the percentage of county residents that did not self-report as White based on the 2017 American Communities Survey^18^), (2) rurality (defined as the percentage of county residents residing in a rural area based on 2010 US Census Bureau data^19^), (3) fine particulate matter (PM_2.5_) concentration (defined as the annual mean county-level PM_2.5_ concentration [measured in micrograms per cubic meter of air] based on 2018 data from the Environmental Protection Agency Environmental Justice Screening tool^20^), (4) county-level median household income (based on 2017 American Community Survey data^18^), (5) high school completion rate (defined as the percentage of county residents ≥25 years that have a high school diploma based on 2015-2019 American Communities Survey data^18^), (6) food insecurity rate (defined as the percentage of county residents that self-reported an inadequate access to food based on 2017 data from Map the Meal Gap^22^), and (7) health care access rate (defined as the number of primary care physicians per 100 000 county residents based on 2017 Area Health Resource Files data^21^) (Table 1).

**Table 1.** Description of the 7 Social and Environmental Determinants of Health Included as Exposures in the Study

Exposure	Detailed description	Year of data collection	Data source
Racial and ethnic minority rate	Percentage of residents in that county that have self-reported themselves identifying as a race or ethnicity other than White	2017	American Communities Survey^18^
Rurality	Percentage of residents in that county that reside in an area defined as rural	2010	US Census Bureau^19^
PM_2.5_ concentration	The annual mean atmospheric concentration of PM_2.5_ levels (μg/m^3^) measured in the county	2018	Environmental Protection Agency^20^
Median household income	The median value of the annual household income measured at the county level for all residents	2017	American Communities Survey^18^
High school completion rate	Percentage of people aged ≥25 y with a high school diploma or equivalent education	2015-2019	American Communities Survey^18^
Food insecurity rate	The percentage of residents in that county who lack adequate access to food	2017	Map the Meal Gap^22^
Primary health care access rate	The number of primary care physicians per 100 000 people in that county.	2017	Area Health Resource Files^21^

Abbreviation: PM_2.5_, fine particulate matter air pollution (<2.5 µg/m^3^).

### County-Level CKM Mortality Rates: CDC WONDER

We defined CKM from the AHA position statement^13^ as the presence of any of the following clinical conditions: cardiac disease (atherosclerotic cardiovascular or cerebrovascular disease, heart failure, or atrial fibrillation), kidney disease (chronic kidney disease defined as an estimated glomerular filtration rate <60 mL/min/1.73 m^2^), metabolic syndrome, obesity (defined as a body mass index >30 [calculated as weight in kilograms divided by height in meters squared]), and diabetes. Using the US Centers for Disease Control and Prevention (CDC) Wide-Ranging Online Data for Epidemiologic Research (WONDER) data portal^1^, we obtained the pooled county-level age-adjusted all-cause mortality rate (aaMR) per 100 000 residents for CKM from January 1, 2010, to December 31, 2019, by entering the relevant *International Statistical Classification of Diseases and Related Health Problems, Tenth Revision (ICD-10)* codes for any of the aforementioned conditions in the multiple causes of death query field (eTable 2 in Supplement 1). Keeping the underlying cause of death field blank to collect aaMR, we limited the age on death certificates to older than15 years to only obtain data on adult residents. The CDC WONDER portal provides age-adjusted rates in 10-year age brackets; therefore, we could not select 18 years as the minimum cohort age. The online CDC WONDER portal compiles results directly from death certificates and was developed by the CDC to specifically disseminate health data to public health officials and researchers.^1^ We limited results to 2019 to exclude any potential impact that the COVID-19 pandemic may have had on the mortality rates. The CDC WONDER portal automatically suppresses information for counties when the age-adjusted death rates are very small (typically <10/100 000 residents); therefore, these data were treated as missing, and these counties were excluded from our analyses.

### Statistical Analysis

We reported the county-level aaMR rates in the US with the median and IQR. We then grouped the aaMR as tertiles and mapped them. We grouped counties according to the US Census Regions (South, West, Midwest, and Northeast). We also grouped counties according to their state. For each state, we calculated the number of counties that belonged to each tertile and ranked the states based on the percentage of counties in the high tertile. We initially fitted a multivariable linear regression model with the aaMR as the outcome and the studied SEDoH as the exposures. For this regression model, we fitted all exposures as continuous variables. From this regression model, we obtained the coefficients for each SEDoH and tested them for statistical significance at the 95% confidence level. The results of this model provide us with the information regarding which SEDoH are associated with the CKM-related aaMR in the US. However, to then explore the spatial heterogeneity in this association, we further fitted a geographically weighted linear regression model.^23^ Unlike the ordinary least squares linear regression models, geographically weighted linear regression also considers the distance between counties and models that distance using weights. These weights change the coefficients observed in the ordinary least squares regression model and provide a separate coefficient for each county. Hence, this set of coefficients obtained from the geographically weighted linear regression model reports what exposures are associated with the outcome for each county. This set of coefficients was also tested at the 95% confidence level to determine which SEDoH were statistically significant for each county. We further analyzed this model to obtain the *R^2^* for each covariate included in the model. The higher the *R^2^*, the more important this variable is to the overall model; hence, we ranked our covariates using this approach. We then compared both models using the *R^2^* value for each model, with a higher *R^2^* depicting a better fit. We also compared the Akaike Information Criterion (AIC) with a lower AIC denoting better predictive accuracy. Unlike the global model that provides a single summary coefficient for each independent variable entered in the model, the geographically weighted regression model provides a separate coefficient at the individual county level for each dependent variable included the model. We examined these coefficients to identify the differential association of counties with the studied SEDoH. In geographically weighted models, the coefficients need to be considered along with their respective *P* values because each county receives its own coefficient for each exposure. Therefore, we obtained county-level *P* values for each county for each exposure using the 2-tailed 95% confidence level. We grouped the *P* values as greater than .05, .05 to .01, and less than .01. We presented both county coefficients and corresponding *P* values as maps. We analyzed our data with R version 4.2.2 (The R Foundation for Statistical Computing) and used the sf, GWmodel packages to fit the geographically weighted models. More details regarding statistical methodology and R packages used is provided in eMethods in Supplement 1. Data analysis was conducted from Month September 2023 to January 2024.

## Results

### Overview of SEDoH

**Figure 1.**
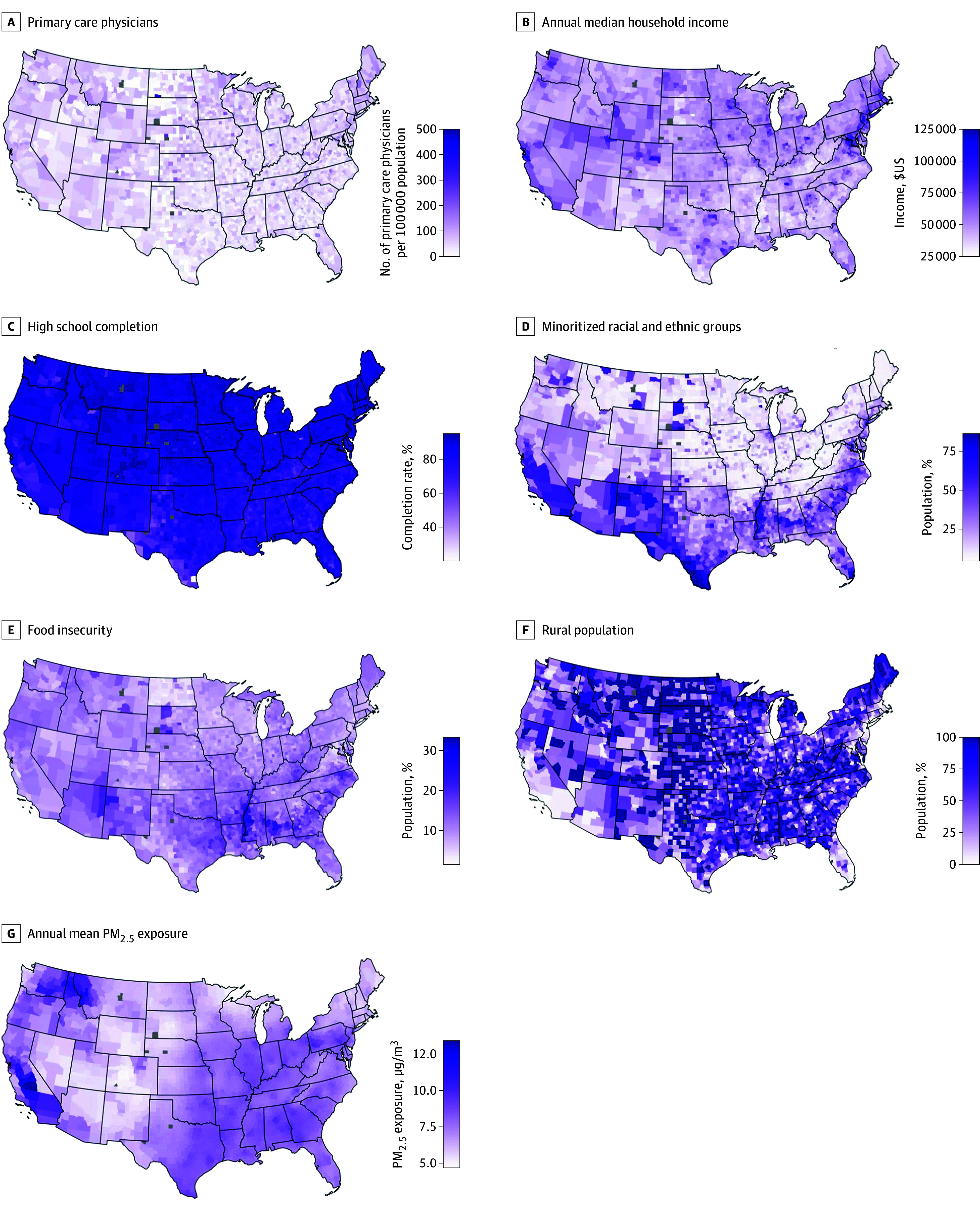
Social and Environmental Determinants of Health Included in the Study In this panel of maps, we presented the county-level values for the 7 social and environmental determinants of health across the US. Gray colored counties indicate counties where data is not available. Readers should evaluate these maps in conjunction with eFigures 1 to 7 in Supplement 1 that report these observations for each US State and Washington, DC. PM_2.5_ indicates fine particulate matter air pollution (<2.5 µg/m3).

In our study, we analyzed data from 3101 of 3243 counties in the US (95.6% of all counties). Across the US, the county-level annual median (IQR) household income was $48 711 ($42 226-$56 539). A large proportion of counties in the South (563 of 1244 counties [45.3%]) were in the low tertile for median household income, while most of the counties in the West (432 of 621 counties [69.5%]) as well as those in the Northeast (135 of 215 counties [62.7%]) were in the middle or highest tertile for annual household income. All counties in Rhode Island and Delaware belonged to the high tertile for median annual household income, while a large proportion of counties in Arkansas (61 of 75 counties [81.3%]) and Mississippi (67 of 82 counties [81.7%]) belonged to the low tertile (Figure 1A). The median (IQR) county-level food insecurity rate across the US was 12.8% (10.6%-15.2%). Again, as with median household income, many counties in the South (575 of 1244 counties [46.2%]) belonged to the high tertile for food insecurity rate. No counties in New Hampshire and Massachusetts belonged to the high tertile, while many counties in Arkansas (64 of 75 [85.3%]) and Mississippi (73 of 84 counties [89.0%]) belonged to the high tertile for food insecurity (Figure 1B and eFigure 1 in Supplement 1). The median (IQR) county-level primary health care access rate was 46.4 (28.9-69.2) primary care physicians per 100 000 county residents. Health care access rates were high in counties located in the West, and Northeast. A large band of counties located in the Southern part of the US were in the low tertile for health care access rates (485 of 1244 [38.9%]). Oklahoma (34 of 77 counties [44.1%]), Mississippi (41 of 82 counties [50.0%]), and Tennessee (39 of 55 counties [41.0%]) had many counties with low primary health care access rates while most counties in Maine (15 of 16 counties [93.7%]) and all counties Rhode Island (10 of 10 counties [100.0%]) and New Hampshire (5 of 5 counties [100.0%]) belonged to the high tertile (Figure 1C and eFigure 2 in Supplement 1). The median (IQR) annual county-level PM_2.5_ concentration was 8.0 (6.8-8.7) μg/m3. Most counties in the Midwest (402 of 1021 counties [39.3%]) were in the low tertile. Many counties in the Northeast (97 of 215 counties [45.1%]) were also in the low tertile for PM_2.5_. Alabama, Illinois, and Arkansas were among the states with the most counties in the high tertile for PM_2.5_ while Minnesota, Massachusetts, Maine, Connecticut, and Arizona had many counties in the low tertile group (Figure 1D and eFigure 3 in Supplement 1). The median (IQR) county-level racial and ethnic minority rate was 16.0% (7.4%-35.0%). Almost one-half of the counties in the Midwest (509 of 1021 counties [49.8%]) were in the low tertile for racial and ethnic minority rates almost one-half of the counties in the South (595 of 1244 counties [47.8%]) were in the high tertile. A large proportion of counties in New Mexico (32 of 33 counties [96.9%]), South Carolina (40 of 46 counties [86.9%]), and Arizona (13 of 15 counties [86.6%]) belonged to the high tertile while Vermont (13 of 14 counties [92.8%]), New Hampshire (9 of 10 counties [90.0%]) and Maine (15 of 16 counties [93.7%]) had most counties belonging to the low tertile (Figure 1E and eFigure 4 in Supplement 1). The county-level median (IQR) rurality rate was 59% (33%-86%). Unlike other studied exposures, rurality appeared to be spread out across the US, and it did not appear to be concentrated in any specific US region. Only Rhode Island and Delaware had counties that belonged only to the low tertile for rurality (Figure 1F and eFigure 5 in Supplement 1). The median (IQR) high school completion rate across the US was 88% (83%-91%). Most counties in Mississippi (68 of 72 counties [76.1%]) and Alabama (51 of 67 counties [76.1%]) belonged to the low tertile for the high school completion rate, while Massachusetts, Delaware, and Connecticut had all counties belonging to either the middle or high tertile for high school completion rate (Figure 1G and eFigure 6 and eFigure 7 in Supplement 1). See eTable 3 in Supplement 1 for more information on SEDoH values by tertile.

### CKM-Related aaMR

**Figure 2.**
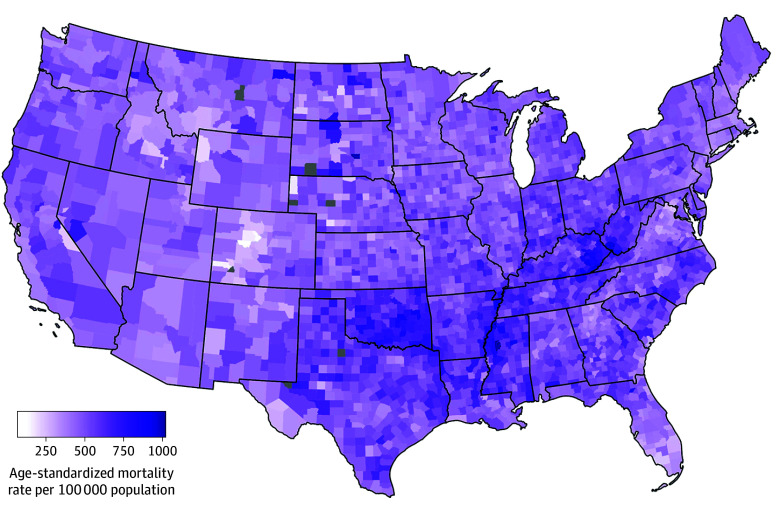
County-Level Cardio-Kidney-Metabolic Syndrome–Attributable All-Cause Mortality Across the US Gray colored counties indicate counties where data is not available.

Of the 3101 included counties, the pooled median (IQR) aaMR (2010-2019) in the US was 505.5 (441.3-578.9) per 100 000 residents (Figure 2). Most counties in the Southern US had rates much higher than the pooled median (eg, Southern US median [IQR] aaMR, 537.3 [466.0-615.9]). Texas, Oklahoma, Louisiana, Alabama, and Georgia were the states with the highest proportion of counties in the high tertile for CKM-related aaMR, while states in the Midwest and West like Minnesota, Wisconsin, Idaho, North Dakota, South Dakota, Wyoming, Montana, as well as those in the West like New Mexico and Arizona, were in the low tertile for CKM-related aaMR.

### Association of CKM Mortality With SEDoH

From the global regression model, we observed that higher CKM aaMR was associated with reduced median household income (β = −0.002; 95% CI, −0.002 to −0.002), reduced primary health care access rate (β = −0.24; 95% CI, −0.34 to −0.14), and high school completion rate (β = −4.39; 95% CI, −5.09 to −3.68) (Table 2). Higher CKM aaMR mortality was also associated with increased food insecurity rate (β = 8.82; 95% CI, 7.65 to 9.99) and PM_2.5_ concentration (β = 7.75; 95% CI, 5.24 to 10.26) (Table 2). From the global model, we observed that median household income, food insecurity, and high school completion rate were ranked as the most important exposures in the model (eTable 4 in Supplement 1).

**Table 2.** Comparing the Geographically Weighted Linear Regression and Ordinary Least Squares Linear Regression Models^a^

Variable	Ordinary least squares regression model		Geographically weighted linear regression model, β, median (IQR)
	β (95%CI)	*P* value	
Annual median household income	−0.002 (−0.002 to −0.002)	<.001	−0.002 (−0.003 to −0.001)
Food insecurity rate	8.82 (7.65 to 9.99)	<.001	6.78 (2.78 to 11.56)
Primary health care access rate	−0.24 (−0.34 to −0.14)	<.001	−0.18 (−0.35 to 0.07)
PM_2.5_ concentration	7.75 (5.24 to 10.26)	<.001	5.52 (−11.06 to 19.70)
Racial and ethnic minority rate	−1.11 (−1.33 to −0.90)	<.001	−0.66 (−1.85 to 0.89)
Rurality rate	−0.26 (−0.39 to −0.14)	<.001	−0.32 (−0.67 to 0.02)
High school completion rate	−4.39 (−5.09 to −3.68)	<.001	−1.89 (−4.54 to 0.10)

Abbreviation: PM_2.5_, fine particulate matter air pollution (<2.5 µg/m^3^).

^a^ This table presents the comparison of model parameters for the ordinary least squares linear regression model (*R^2^* = 41.1%; Akaike information criterion-corrected = 36 233) and the geographically weighted linear regression model (*R^2^* = 69.4%; Akaike information criterion-corrected = 35 110).

CKM-related mortality was positively associated with the food insecurity rate (median [IQR] β = 6.78 [2.78-11.56]) and PM_2.5 _concentrations (median [IQR] β = 5.52 [−11.06 to 19.70]). CKM-related mortality was negatively associated with median annual household income (median [IQR] β = −0.002 [−0.003 to −0.001]), rurality (median [IQR] β = −0.32 [−0.67 to 0.02]), high school completion rate (median [IQR] β = −1.89 [−4.54 to 0.10]), racial and ethnic minority rate (median [IQR] β = −0.66 [−1.85 to 0.89]), and primary health care access rate (median [IQR] β = −0.18 [−0.35 to 0.07]).

**Figure 3.**
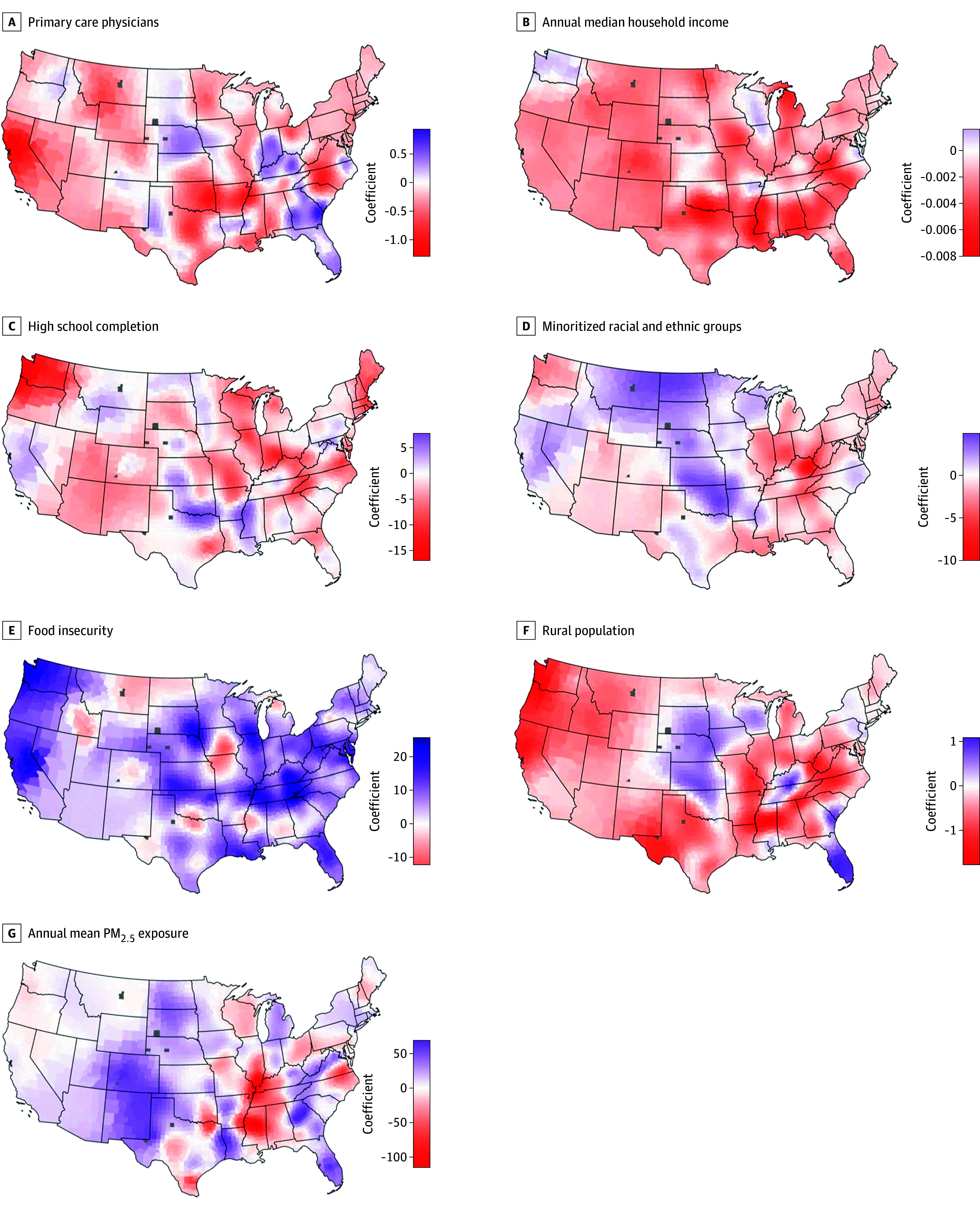
Coefficients for Each Social and Environmental Determinant of Health Included in the Geographically Weighted Regression Model In this panel of maps, we present the results of the multivariable geographically weighted linear regression model fitted to explore the association of county-level cardio-kidney-metabolic syndrome–related all-cause mortality rate and the studied social and environmental determinants of health. We have presented the coefficient for each county for each exposure included in our geographically weighted linear regression model. Gray colored counties indicate counties where data is not available. These maps should be studied along with the *P* values reported for each county for each exposure, which are presented in eFigure 8 in Supplement 1.

Therefore, from the geographically weighted model, we observed that the magnitude of the association (as measured by the *P* values) varied between counties in the US. First, we observed that the geographically weighted model was better than the global model (higher *R^2^* value and lower AIC value). Most counties in the US reported an *R^2^* of 75% or greater (Figure 3A). We observed that the studied exposures were important in different regions of the US (Figure 3, B-H). Median household income, PM_2.5_ concentration, and racial and ethnic minority rate were important for a large band of counties across the Midwest (see eFigure 8 in Supplement 1 for more detail by county). Median household income was also important for counties in the South. However, primary health care access rate appeared to be very important for only a few counties in Montana and Wyoming.

## Discussion

### Salient Findings

In this cross-sectional study analyzing county-level data to assess the differential association of CKM aaMR and SEDoH across the US, we observed that CKM-related mortality was higher in counties with lower median household income, a higher racial and ethnic minority rate, more food insecurity, higher PM_2.5_ exposure, lower high school completion rates, lower primary care access, and more rural residents. Overall, median household income, food insecurity rate, and high school completion rate were the most important exposures associated with CKM aaMR. The geographically weighted model further exposed the variable magnitude of the association of the studied exposures with CKM-related aaMR in the US.

### Public Health Importance

The World Health Organization defines social determinants of health as “the conditions in which people are born, grow, work, live and age, and the wider set of forces and systems shaping the conditions of daily life.^24^” Therefore, by its very definition, SEDoH are factors that are known to vary across regions. It is therefore logical that they should be evaluated using models that take this fact into account. However, most studies that have studied the epidemiology of cardiovascular mortality rates across the US have used the included exposures as a fixed effect.^10,25^ Our study, thus, supports prior evidence by confirming the direction of the association of SEDoH with CKM mortality and ranking these exposures according to their importance overall in the US. However, we go beyond this by demonstrating that the magnitude of the association of SEDoH and cardiovascular mortality in the US varied across the US. We believe that our study results should be considered when deciding public health policy at the local level. In fact, our study also demonstrates that when accounting for multiple SEDoH together in a single model, the magnitude of the association of exposure with outcome may be independent of the level of that exposure. For example, while many states across the lower half of the US have a large proportion of counties that were in the high tertile for food insecurity rate, our map demonstrates that median household income appears to be the most important factor associated with CKM-related aaMR in this region. Therefore, the wider use of geographically weighted models will allow us to understand and choose those SEDoH exposures that need to be addressed more urgently at the local level. The public health care system in the US has been perennially underfunded, with allocated money remaining static for the past decade.^26^ Therefore, studies such as ours are needed to understand local factors associated with cardiovascular outcomes in the community. In our study, we have also included factors such as the county level racial and ethnic composition, which may not change much over time. While these factors are also important at the individual level, at the population health level, we must focus our attention on those health and social factors that can be improved over time. To date, the use of geographically weighted models to study cardiovascular disease is very limited, and we hope that researchers will use these tools to model more flexible associations of exposures with disease^27,28,29^.

### Limitations and Strengths

Readers should understand our results on the background of specific study limitations. First, because this is a study based on summary county-level information, there is the possibility of ecological fallacy, and there are some specific limitations associated with the use of the CDC WONDER data that we would like to highlight. The CDC WONDER automatically replaced result values smaller than 10 per 100 000 residents as missing, and therefore we included 95.6% of all US counties. Additionally, we identified CKM using *ICD-10* codes from death certificate data and were unable to evaluate the stages of CKM. The AHA statement^13^ reported that CKM is a very heterogenous phenotype and outcomes will vary depending upon the stage of disease. While we feel that future work should evaluate our results according to CKM stage, the nationwide perspective that we have been able to provide will not be possible with patient-level cohort data. Second, given the aggregate nature of our data, we used publicly available SEDoH measurements linked at the county level; the SEDoH will vary between individuals in a single geographical unit, and our study cannot account for that. However, we provide important population-level inferences, and public policy is often decided based on potential benefit for the whole community rather than a few individuals. Furthermore, if certain public health efforts like improving air quality and better (and free) primary health care coverage are provided, even people that are not socially deprived will stand to benefit. Additionally, we used many different SEDoH exposures, some of which may be collinear; however, studies have reported that geographical weighting removes this modeling issue and provides unbiased estimates. This fact is also evident from the improved model metrics that we have reported in our study. Some reported areas of debate regarding geographically weighted models are how to select appropriate weights, use of the model for explanation vs inference, and defining the degrees of freedom. While some consider multicollinearity to be an issue, the opinion is divided.^30,31^

## Conclusions

In conclusion, in this cross-sectional study, we fit geographically weighted models to investigate the differential association of county-level SEDoH and CKM-related mortality in the US. We observed that the overall burden of CKM was high, with wide differences across counties in the magnitude of the association of studied exposures with CKM mortality. Future work may be directed toward better understanding the impact of these SEDoH on the various stages of CKM.

## Notes

Supplement 1. **eTable 1.** Different Exposures Considered for Inclusion in Our Study **eTable 2.** *ICD* Codes Used to Define CKM
eMethods.
**eFigure 1.** Distribution of the County-Level Food Insecurity Rate Tertile in Each US State **eFigure 2.** Distribution of the Primary Healthcare Access Rate Tertile in Each US State **eFigure 3.** Distribution of the Air Pollution Concentration Tertile in Each US State **eFigure 4.** Distribution of the Race/Ethnic Minority Rate in Each US State **eFigure 5.** Distribution of the Rurality Rate in Each US State **eFigure 6.** Distribution of the High School Completion Rate in Each US State **eFigure 7.** Cardio-Kidney-Metabolic Syndrome Related Mortality Rates for Each US State **eTable 3.** Tertile Values for Each SEDoH **eTable 4.** Relative Importance of Exposures **eFigure 8.** Map of R^2^ and *P*-Values From the Model

Supplement 2.
Data Sharing Statement
