# Supplementary material for: Social and Environmental Determinants of Health and Cardio-Kidney-Metabolic Syndrome–Related Mortality
Source: JAMA Netw Open. 2024 Sep 26;7(9):e2435783. doi: 10.1001/jamanetworkopen.2024.35783 (PMC11427959; doi:10.1001/jamanetworkopen.2024.35783)
Supplement: Supplement 1. — eTable 1. Different Exposures Considered for Inclusion in Our Study eTable 2. ICD Codes Used to Define CKM eMethods. eFigure 1. Distribution of the County-Level Food Insecurity Rate Tertile in Each US State eFigure 2. Distribution of the Primary Healthcare Access Rate Tertile in Each US State eFigure 3. Distribution of the Air Pollution Concentration Tertile in Each US State eFigure 4. Distribution of the Race/Ethnic Minority Rate in Each US State eFigure 5. Distribution of the Rurality Rate in Each US State eFigure 6. Distribution of the High School Completion Rate in Each US State eFigure 7. Cardio-Kidney-Metabolic Syndrome Related Mortality Rates for Each US State eTable 3. Tertile Values for Each SEDoH eTable 4. Relative Importance of Exposures eFigure 8. Map of R2 and P-Values From the Model [file jamanetwopen-e2435783-s001.pdf]

Supplemental Online Content

Vieira de Oliveira Salerno PR, Cotton A, Elgudin YE, et al. Social and environmental determinants of health and cardio-kidney-metabolic ckm syndrome–related mortality. *JAMA Netw. Open*. 2024;7(9):e2435783. doi:10.1001/jamanetworkopen.2024.35783

eTable 1. Different Exposures Considered for Inclusion in Our Study

eTable 2. *ICD* Codes Used to Define CKM

eMethods.

eFigure 1. Distribution of the County-Level Food Insecurity Rate Tertile in Each US State

eFigure 2. Distribution of the Primary Healthcare Access Rate Tertile in Each US State

eFigure 3. Distribution of the Air Pollution Concentration Tertile in Each US State

eFigure 4. Distribution of the Race/Ethnic Minority Rate in Each US State

eFigure 5. Distribution of the Rurality Rate in Each US State

eFigure 6. Distribution of the High School Completion Rate in Each US State

eFigure 7. Cardio-Kidney-Metabolic Syndrome Related Mortality Rates for Each US State

eTable 3. Tertile Values for Each SEDoH

eTable 4. Relative Importance of Exposures

eFigure 8. Map of R^2 and *P*-Values From the Model

This supplemental material has been provided by the authors to give readers additional information about their work.

© 2024 Vieira de Oliveira Salerno PR et al. *JAMA Network Open.*

eTable 1. Different Exposures Considered for Inclusion in Our Study

**Race/Ethnicity and Population Structure**	**Description**	**Year**	**Original Source**	**Race/Ethnicity and Population Structure**
Hispanic Population	Percentage of population identifying as Hispanics	2017	Census -PE	Hispanic Population
Non-Hispanic White	Percentage of Non-Hispanic White population	2017	Census -PE	Non-Hispanic White
Non-Hispanic Black	Percentage of Non-Hispanic African American population	2017	Census -PE	Non-Hispanic Black
Asian and Pacific Islander	Percentage of Asian and Pacific Islander population	2017	Census -PE	Asian and Pacific Islander
Female Population	Percentage of female population	2017	Census -PE	Female Population
Rural Population	Percentage of people living in rural areas	2010	Census -PE	Rural Population
Population above 65 years	Percentage of population age 65 years or older	2017	Census -PE	Population above 65 years
Population under 18 years	Percentage of population age 18 years or younger	2017	Census -PE	Population under 18 years
**Environmental Exposure**				**Environmental Exposure**
Particulate Matter 2.5 Level in Air	$PM_{2.5}$ levels in air, µg/m3 (annual average)	2018	EPA - EJSCREEN	Particulate Matter 2.5 Level in Air
Diesel Particulate Matter Level in Air	Diesel particulate matter level in air, µg/m3	2017	EPA- EJSCREEN	Diesel Particulate Matter Level in Air
Ozone Level in Air	Ozone summer seasonal average of daily maximum 8 h concentration in air in parts per billion	2018	EPA- EJSCREEN	Ozone Level in Air
Air Toxics Cancer Risk	Lifetime exposure to air toxics cancer risk (in persons per million)	2017	EPA- EJSCREEN	Air Toxics Cancer Risk
Proximity to NPL Sites	Count of proposed or listed NPL—also known as superfund—sites within 5 km (or nearest one beyond 5 km), each divided by distance in km	2020	EPA - EJSCREEN	Proximity to NPL Sites
Proximity to RMP Sites	Count of RMP (potential chemical accident management plan) facilities within 5 km (or nearest one beyond 5 km), each divided by distance in km	2020	EPA - EJSCREEN	Proximity to RMP Sites

© 2024 Vieira de Oliveira Salerno PR et al. *JAMA Network Open.*

Traffic Proximity and Volume	Average annual daily count of vehicles at major roads within 500 m, divided by distance in meters	2019	EPA - EJSCREEN	Traffic Proximity and Volume
**Health Behaviors and Conditions**				**Health Behaviors and Conditions**
Adult Smoking	Percentage of adults who are current smokers (age-adjusted)	2017	CDC - BRFSS	Adult Smoking
Obesity	Percentage of the adult population (aged ≥18 y) that reports a body mass index ≥30 (age-adjusted)	2017	CDC - DSS	Obesity
Diabetes	Percentage of adults (age ≥ 20) with diagnosed diabetes (age-adjusted)	2017	CDC - DSS	Diabetes
Low Birthweight	Percentage of live births with low birthweight (< 2,500 grams)	2013-2019	CDC - NCHS	Low Birthweight
Flu Vaccinations	Percentage of fee-for-service Medicare enrollees that had an annual flu vaccination	2017	CMS -MMD	Flu Vaccinations
Excessive Drinking	Percentage of adults reporting binge or heavy drinking (age-adjusted)	2017	CDC - BRFSS	Excessive Drinking
Access to Exercise Opportunities	Percentage of people with adequate access to locations for physical activity	2010, 2019	ESRI & Census -TF	Access to Exercise Opportunities
Physically Inactive	Percentage of adults (age ≥ 18 y) reporting no leisure-time physical activity (age-adjusted)	2017	CDC- DSS	Physically Inactive
Poor Mental Health Days	Average number of mentally unhealthy days reported in past 30 days (age-adjusted).	2019	CDC - BRFSS	Poor Mental Health Days
Insufficient Sleep	Percentage of adults who report fewer than 7 hours of sleep on average (age-adjusted).	2018	CDC - BRFSS	Insufficient Sleep
Poor Physical Health Days	Average number of physically unhealthy days reported in past 30 days (age-adjusted).	2019	CDC - BRFSS	Poor Physical Health Days
Frequent Mental Distress	Percentage of adults reporting 14 or more days of poor mental health per month (age-adjusted).	2019	CDC - BRFSS	Frequent Mental Distress
Frequent Physical Distress	Percentage of adults reporting 14 or more days of poor physical	2019	CDC - BRFSS	Frequent Physical Distress

© 2024 Vieira de Oliveira Salerno PR et al. *JAMA Network Open.*

	health per month (age-adjusted).			
**Socioeconomic Factors**				**Socioeconomic Factors**
Median Household Income	The income (US dollar) where half of households in a county earn more and half of households earn less	2017	AHRF	Median Household Income
Unemployment	Percentage of people (aged≥16 year) unemployed but seeking work	2017	BLS	Unemployment
Income Inequality	Ratio of household income at the 80th percentile to income at the 20th percentile	2015-2019	Census - ACS	Income Inequality
Poverty	Percentage of people whose income under the federal poverty level	2017	AHRF	Poverty
Under 200% Poverty (18-64 years)	Percentage of people (aged 18–64 year) whose income is under 200% of the federal poverty level	2017	AHRF	Under 200% Poverty (18-64 years)
Children in Poverty	Percentage of people under age 18 in poverty.	2020	Census - SAIPE	Children in Poverty
High School Completion	Percentage of people aged ≥ 25 years with a high school diploma or equivalent	2015-2019	Census - ACS	High School Completion
Some College Degree	Percentage of people (aged 25–44 year) with some post-secondary education	2015-2019	Census - ACS	Some College Degree
Severe Housing Problems	Percentage of households with at least 1 of 4 housing problems: overcrowding, high housing costs, lack of kitchen facilities, or lack of plumbing facilities	2013-2017	CHAS	Severe Housing Problems
Severe Housing Cost Burden	Percentage of households that spend 50% or more of their household income on housing	2015-2019	Census-ACS	Severe Housing Cost Burden
Homeownership	Percentage of owner-occupied housing units	2015-2019	Census - ACS	Homeownership
Children in Single-Parent Household	Percentage of children that live in a household headed by a single parent.	2016-2020	Census - ACS	Children in Single-Parent Household
Limited Access to Healthy Food	Percentage of people who are low-income and do not live close to a grocery store	2015	USDA -FEA	Limited Access to Healthy Food

© 2024 Vieira de Oliveira Salerno PR et al. *JAMA Network Open.*

Food Stamp Recipients	Percentage of people who were food stamp recipients	2017	AHRF	Food Stamp Recipients
Food Insecurity	Percentage of people who lack adequate access to food	2017	MMG	Food Insecurity
Food Environment Index	Index of factors that contribute to a healthy food environment, from 0 (worst) to 10 (best).	2019	USDA - FEA	Food Environment Index
Social Association	Number of membership associations per 10,000 population	2017	CBP	Social Association
Injury Deaths	Number of deaths due to injury per 100,000 population.	2016-2020	NVSS	Injury Deaths
Uninsured Rate	Percentage of people (aged 18–64 year) without health insurance	2017	HRSA - AHRF	Uninsured Rate
Long Commute – Driving Alone	Among workers who commute in their car alone, the percentage that commute more than 30 minutes.	2016-2020	Census -ACS	Long Commute – Driving Alone
Primary Care Physicians	Primary care physicians in patient care per 100,000 people	2017	HRSA -AHRF	Primary Care Physicians
Broadband Access	Percentage of households with broadband internet connection	2015-2019	Census -ACS	Broadband Access

This table provides the different exposures that we considered in our study. Using a combination of expert knowledge, review of prior literature, and current understanding, we eventually chose 7 different SEDoH which we encompass the different SEDoH measures that are known to be strongly associated with CVD

Abbreviations: AHRF: Area Health Resources Files; ACS: American Community Survey; BLS: Bureau of Labor Statistics; BRFSS: Behavioral Risk Factor Surveillance System; CBP: County Business Patterns; CDC: Centers for Disease Control and Prevention; CHAS: Comprehensive Housing Affordability Strategy; CHF: County Health Rankings & Roadmaps; CMS: Centers for Medicare & Medicaid Services; DSS: US Diabetes Surveillance System; EJSCREEN: Environmental Justice Screening tool ; EPA: Environmental Protection Agency; FEA: Food Environment Atlas; HRSA: Health Resources and Services Administration; MMD: Mapping Medicare Disparities (MMD) Tool; MMG: Map the Meal Gap; NCHHSTP: National Center for HIV/AIDS, Viral Hepatitis, STD, and TB Prevention; NCHS: National Center for Health Statistics; NLP: National Priorities List; PE: Population Estimates; PM: Fine particulate matter; RMP: Risk Management Plan; USDA: US Department of Agriculture.

© 2024 Vieira de Oliveira Salerno PR et al. *JAMA Network Open.*

eTable 2. *ICD* Codes Used to Define CKM

**ICD Code**	**Description**
I10-I15	Hypertensive diseases
I20-I25	Ischaemic heart diseases
I60-I69	Cerebrovascular diseases
I70	Atherosclerosis
I48	Atrial fibrillation and flutter
I50	Heart failure
N17-N19	Renal failure
E11	Non-insulin-dependent diabetes mellitus
E13	Other specified diabetes mellitus
E65	Localized adiposity
E66	Obesity
E78	Disorders of lipoprotein metabolism and other lipidaemias

In this table we present the ICD codes used to define CKM according to the American Heart Association statement

© 2024 Vieira de Oliveira Salerno PR et al. *JAMA Network Open.*

eMethods

Statistical Details

Traditional models, like ordinary least square regression, presuppose a consistent relationship between covariates and outcomes throughout the entire studied area. Consequently, these models overlook potential variations in the relationship between outcomes and covariates based on geographical differences within the study area. [1] To account for this non-stationary relationship in space, local models such as Geographically Weighted Regression (GWR) were developed. [1] It is a non-parametric technique that can be expressed by the following formula:

$$y_i = \beta_0(u_i, v_i) + \sum_k \beta_k(u_i, v_i) x_{ik} + \varepsilon_i$$

The coordinates of the i-th point in space are denoted as $\beta_0(u_i, v_i)$, with $\beta_k(u_i, v_i)$ representing the realization of the continuous function at point i.[2] This approach allows for the estimation of coefficients at each data location, and observations are weighted based on their proximity to location i. GWR assumes that observations that are closer to each other have a greater influence than the observations that are farther away.[2] These weights are based on a distance decay function centered on observation i, which is further adjusted by a bandwidth setting at which the distance weight approaches zero. [2] Since there is a regression equation for each observation, calibrated independently, a t-ratio and R^2 can be obtained for each observation.[2]

In our study, we used the R Foundation for Statistical Computing (R) package “spgwr” was used to fit the GWR. First, we used the cross-validation method to identify the kernel bandwidth, adopting a Gaussian weighting function and selecting an adaptive kernel type to account for the variation of the density of observations in the study area. Next, we fit the GWR model using the bandwidth previously identified. The t-ratios obtained (variable coefficient/ variable standard error) from the GWR were converted into two-tailed p values using the pt function in R [2 * pt(-abs(t-ratio), dfree)]. Model fit was assessed using R^2 and Akaike Information Criterion-corrected (AICc).

References:

1. Goovaerts P. Geostatistical Analysis of Health Data: State-of-the-Art and Perspectives. In: Soares A, Pereira MJ, Dimitrakopoulos R, eds. *geoENV VI – Geostatistics for Environmental Applications: Proceedings of the Sixth European Conference on Geostatistics for Environmental Applications*. Quantitative Geology and Geostatistics. Springer Netherlands; 2008:3-22. doi:10.1007/978-1-4020-6448-7_1

2. Thapa RB, Estoque RC. Geographically Weighted Regression in Geospatial Analysis. In: Murayama Y, ed. *Progress in Geospatial Analysis*. Springer Japan; 2012:85-96. doi:10.1007/978-4-431-54000-7_6

R code

Bandwidth selection:

```
bwG2 <- gwr.sel(rate_i ~ PM25 +  Med_H_income + R_RacialEthnic_Minorities + Food_insecurity + R_Pri_Care_Phys + High_school + Rural_R,
        data = df2.sp,
        gweight = gwr.Gauss,
        verbose = TRUE,
        adapt = TRUE)
```

Running the GWR:

```
gwrG2 <- gwr(rate_i ~ PM25 +  Med_H_income + R_RacialEthnic_Minorities + Food_insecurity + R_Pri_Care_Phys + High_school + Rural_R,
       data = df2.sp,
       adapt = bwG2,
```

© 2024 Vieira de Oliveira Salerno PR et al. *JAMA Network Open.*

```
            gweight = gwr.Gauss,
            hatmatrix = TRUE)
```

Obtaining the T-ratios and converting them into p-values:

```
#obtain degrees f freedom
dfree<-gwrG2$results$edf
#calculate t ratio
sf_data$pm.t <- gwrG2$SDF$PM25/gwrG2$SDF$PM25_se
sf_data$food.t <- gwrG2$SDF$Food_insecurity/gwrG2$SDF$Food_insecurity_se
sf_data$race.t <- gwrG2$SDF$R_RacialEthnic_Minorities/gwrG2$SDF$R_RacialEthnic_Minorities_se
sf_data$income.t <- gwrG2$SDF$Med_H_income/gwrG2$SDF$Med_H_income_se
sf_data$pcp.t <- gwrG2$SDF$R_Pri_Care_Phys/gwrG2$SDF$R_Pri_Care_Phys_se
sf_data$rural.t <- gwrG2$SDF$Rural_R/gwrG2$SDF$Rural_R_se
sf_data$school.t <- gwrG2$SDF$High_school/gwrG2$SDF$High_school_se

#calculate p values
sf_data$pm.t.p<-2*pt(-abs(sf_data$pm.t), dfree)
sf_data$food.t.p<-2*pt(-abs(sf_data$food.t), dfree)
sf_data$race.t.p<-2*pt(-abs(sf_data$race.t), dfree)
sf_data$income.t.p<-2*pt(-abs(sf_data$income.t), dfree)
sf_data$pcp.t.p<-2*pt(-abs(sf_data$pcp.t), dfree)
sf_data$rural.t.p<-2*pt(-abs(sf_data$rural.t), dfree)
sf_data$school.t.p<-2*pt(-abs(sf_data$school.t), dfree)
```

Packages used:
1. Geographically weighted models:

sf - Pebesma E, Bivand R (2023). *Spatial Data Science: With applications in R*. Chapman and Hall/CRC. doi:10.1201/9780429459016, https://r-spatial.org/book/.

GWmodel - Isabella Gollini, Binbin Lu, Martin Charlton, Christopher Brunsdon, Paul Harris (2015). "GWmodel: An R Package for Exploring Spatial Heterogeneity Using Geographically Weighted Models." *Journal of Statistical Software*, **63**(17), 1–50.doi:10.18637/jss.v063.i17.

Maps:
usmap - https://cran.r-project.org/web/packages/usmap/index.html

© 2024 Vieira de Oliveira Salerno PR et al. *JAMA Network Open.*

eFigure 1. Distribution of the County-Level Food Insecurity Rate Tertile in Each US State

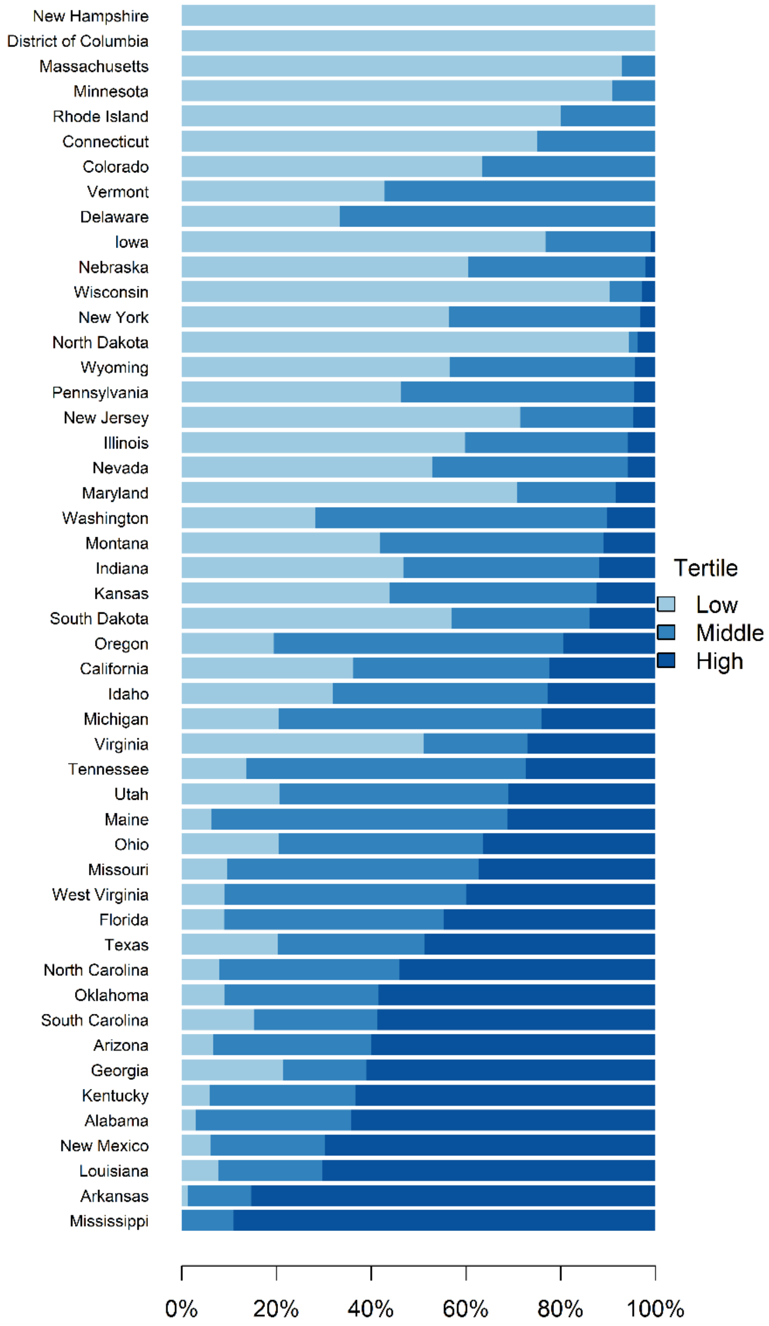

In this bar plot we present the distribution of the tertile for county-level food insecurity rate for all US States. As seen, New Hampshire and the District of Columbia had all counties in the low tertile while a very large proportion of counties in Arkansas and Mississippi were in the high tertile for food insecurity.

© 2024 Vieira de Oliveira Salerno PR et al. *JAMA Network Open.*

eFigure 2. Distribution of the Primary Healthcare Access Rate Tertile in Each US State

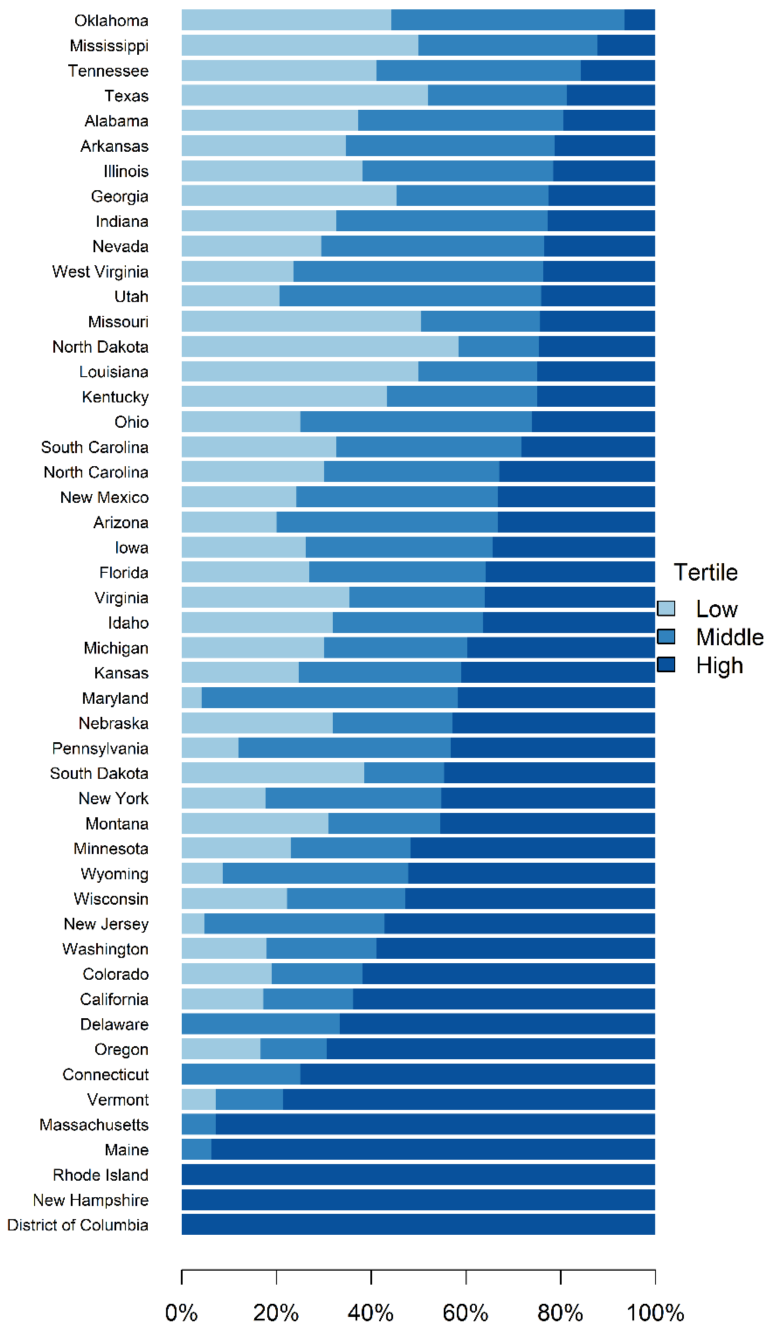

In this bar plot we present the distribution of the tertile for the primary healthcare access rate for all US States. As seen, Oklahoma and Mississippi have many counties in the low tertile for primary healthcare access while Rhode Island, New Hampshire and the District of Columbia had all counties in the high tertile.

© 2024 Vieira de Oliveira Salerno PR et al. *JAMA Network Open.*

eFigure 3. Distribution of the Air Pollution Concentration Tertile in Each US State

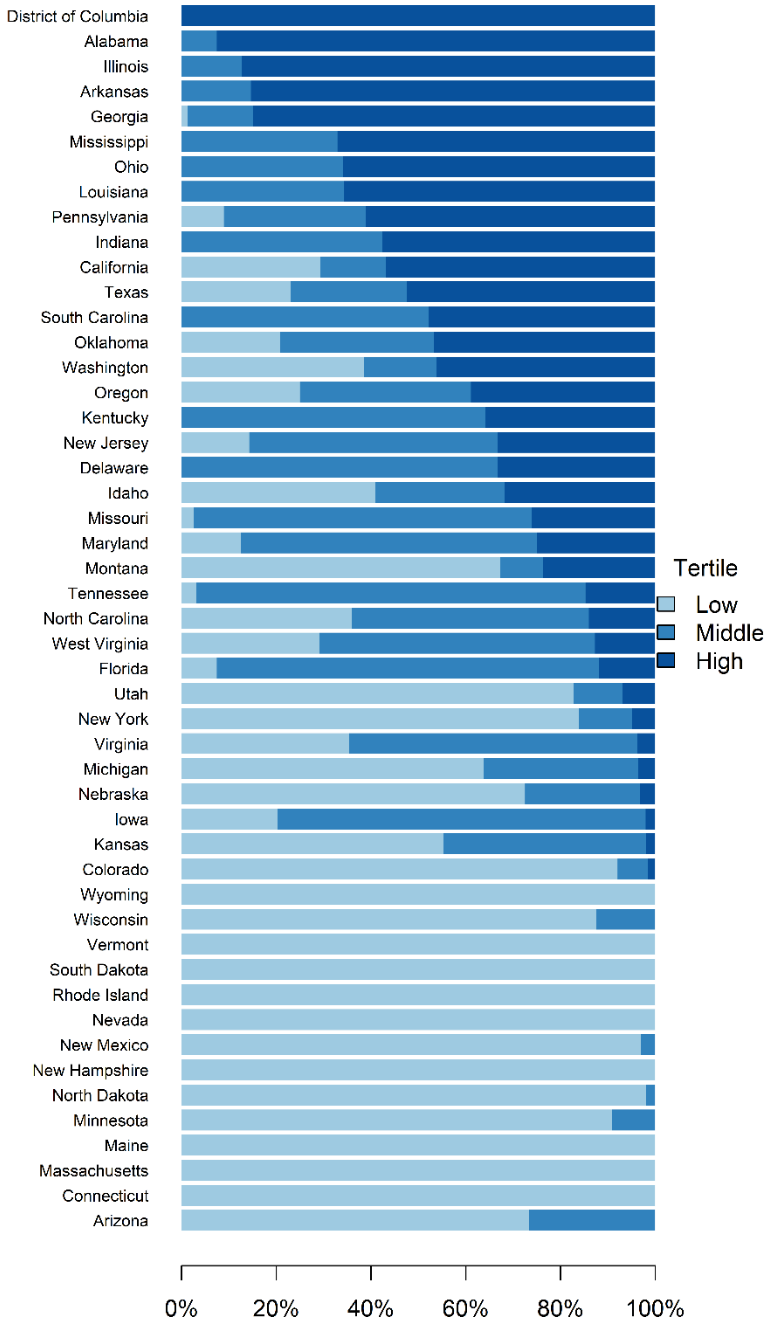

In this barplot, we report the distribution of air pollution ($PM_{2.5}$) tertile that each county belongs to in each US state. As shown here, counties that are in the high tertile for $PM_{2.5}$ are quite distributed among states.

© 2024 Vieira de Oliveira Salerno PR et al. *JAMA Network Open.*

eFigure 4. Distribution of the Race/Ethnic Minority Rate in Each US State

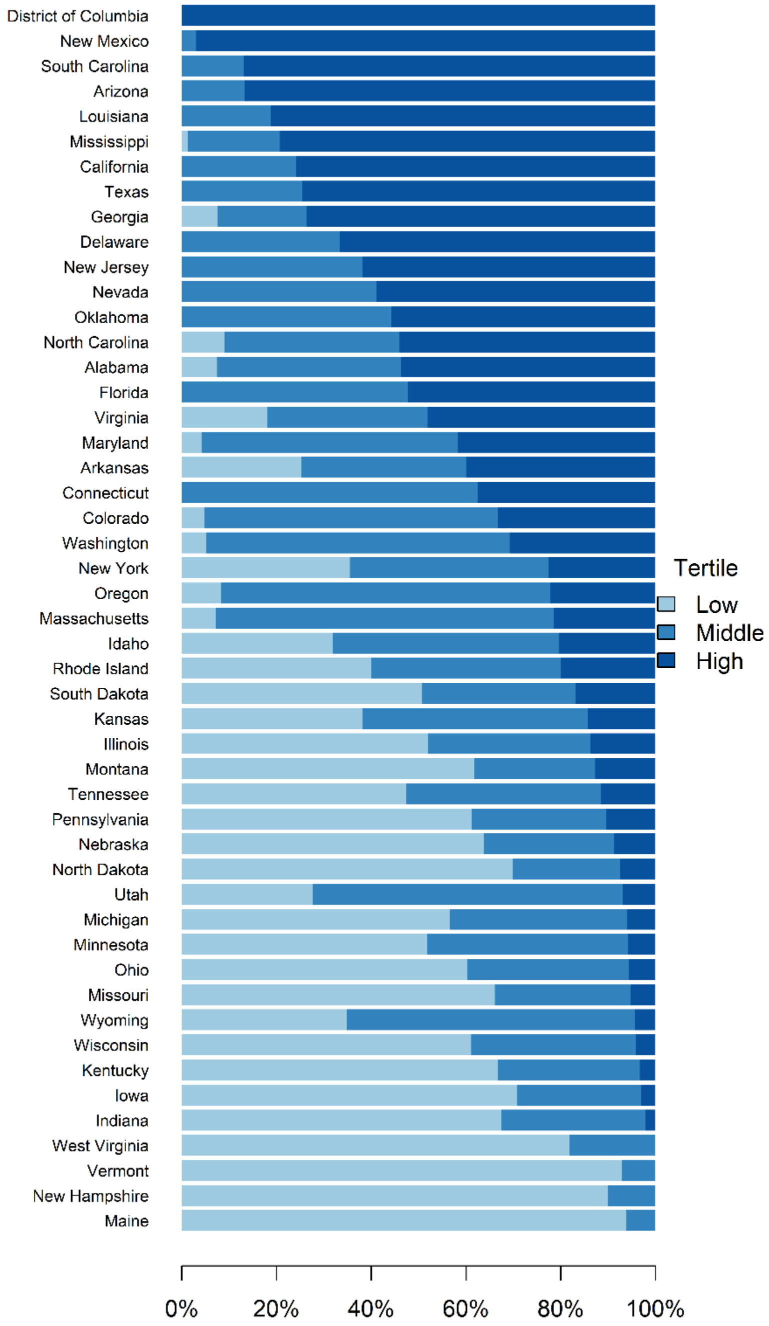

In this barplot, we have presented the tertile for the racial/ethnic minority rate at the county level. The plot depicts the proportion of counties that belong to the low, middle, and high tertile for racial/ethnic minorities in each state. Please note that the District of Columbia is a single county. Hence, New Mexico and South Carolina have a high number of counties in the high tertile while Maine and New Hampshire have many counties in the low tertile.

© 2024 Vieira de Oliveira Salerno PR et al. *JAMA Network Open.*

eFigure 5. Distribution of the Rurality Rate in Each US State

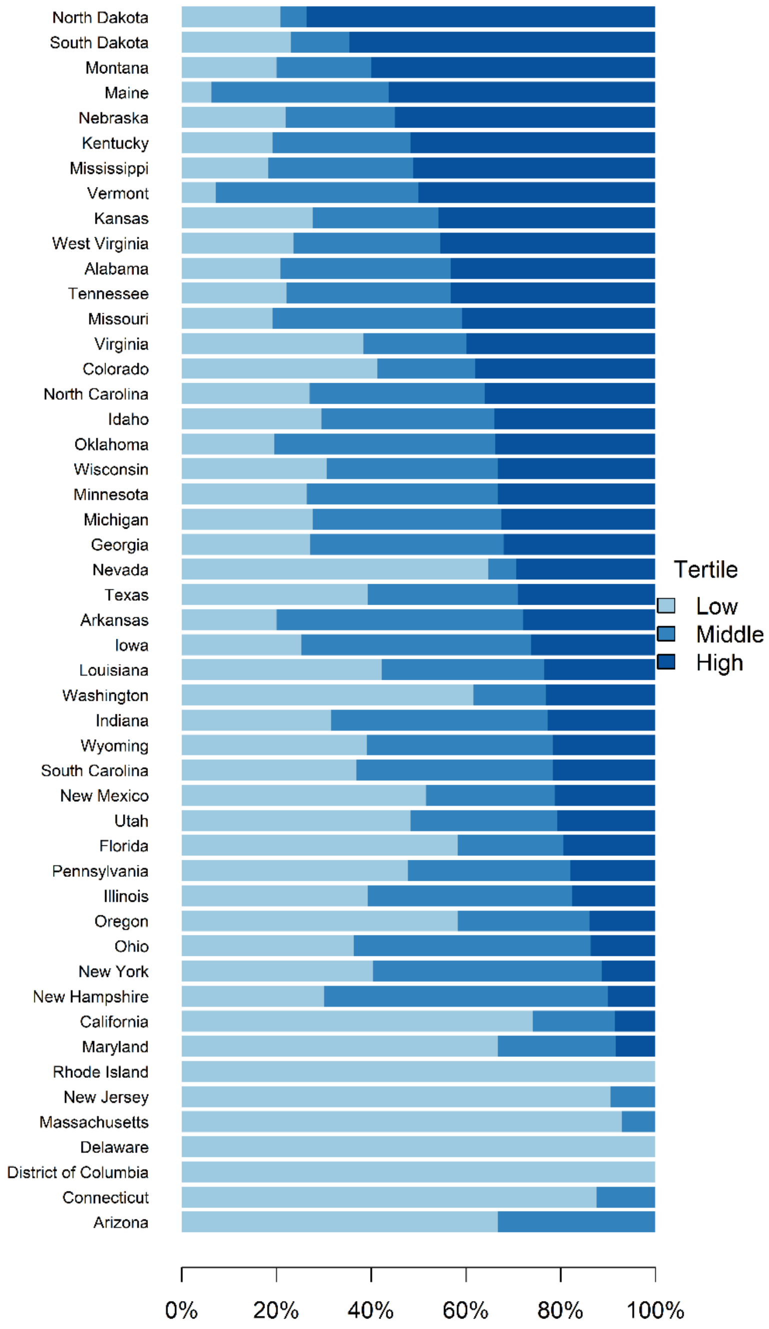

In this barplot, we have presented the tertile for the rurality rate at the county level. The plot depicts the proportion of counties that belong to the low, middle, and high tertile for rurality in each state. North and South Dakota followed by Montana have the most counties in the high tertile for rurality while Connecticut, Arizona, Delaware has many counties in the low tertile for rurality.

© 2024 Vieira de Oliveira Salerno PR et al. *JAMA Network Open.*

eFigure 6. Distribution of the High School Completion Rate in Each US State

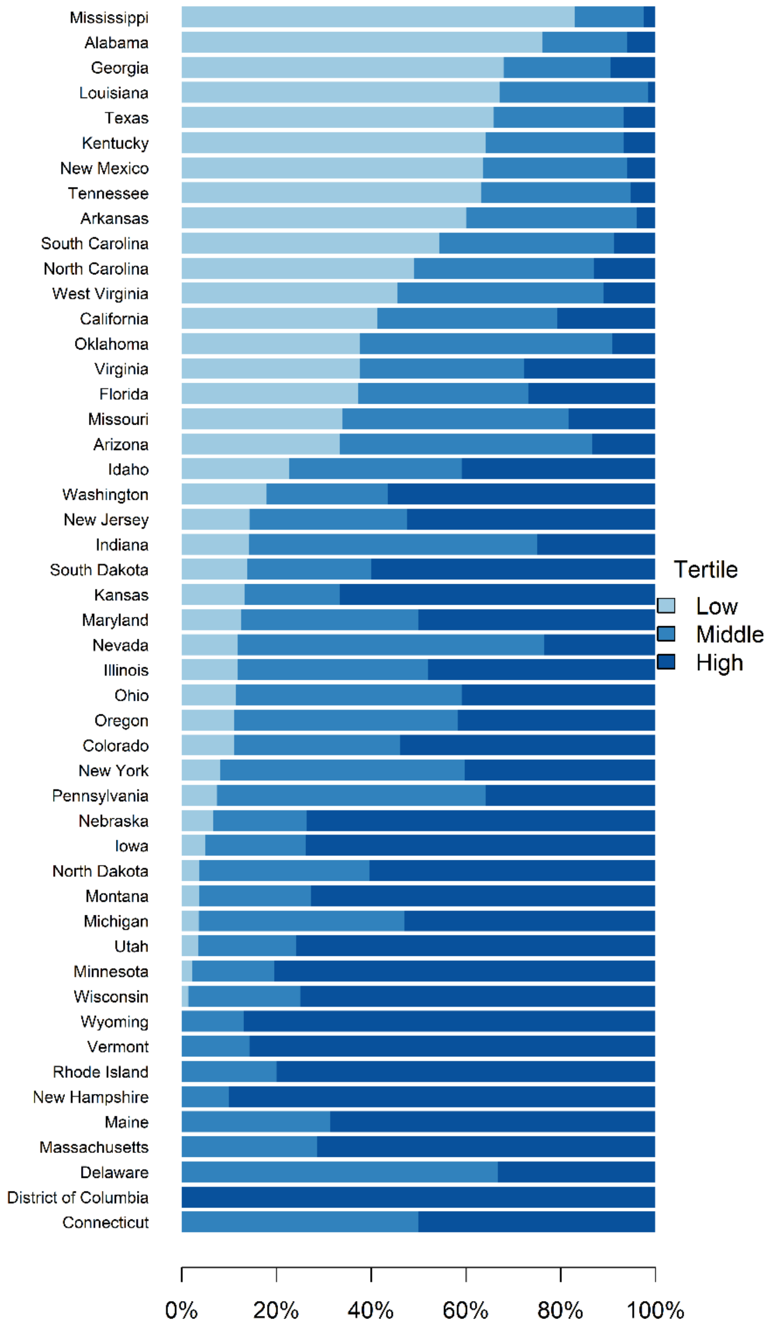

In this barplot, we have presented the tertile for the high school completion rate at the county level. The plot depicts the proportion of counties that belong to the low, middle, and high tertile for high school completion in each state. As shown, Mississippi and Alabama have many counties in the low tertile for high school completion while Connecticut, Delware, New Hampshire all have very many counties in the high tertile for high school completion. Please note that the the District of Columbia is a single county

© 2024 Vieira de Oliveira Salerno PR et al. *JAMA Network Open.*

eFigure 7. Cardio-Kidney-Metabolic Syndrome Related Mortality Rates for Each US State

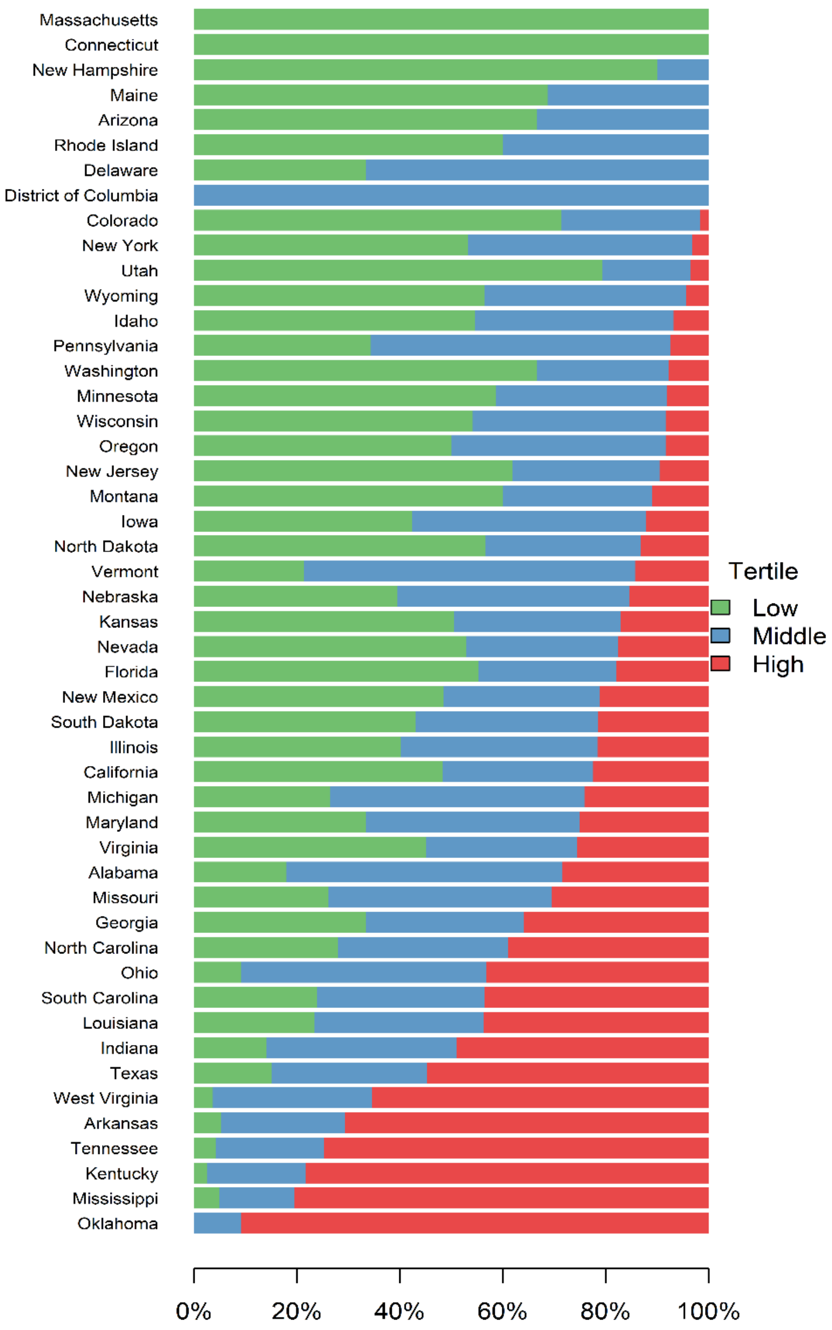

In this bar plot we present the distribution of the tertile for county-level age adjusted all-cause mortality related to cardio-kidney-metabolic syndrome for all US States. As seen, Massachusetts and Connecticut had the best distribution with all counties in the low tertile, while Mississippi and Oklahoma had the worst distribution as a large proportion of counties belonged to the high tertile.

© 2024 Vieira de Oliveira Salerno PR et al. *JAMA Network Open.*

eTable 3. Tertile Values for Each SEDoH

Exposure	Low tertile	Middle tertile	High tertile
Annual median household income	$22 679 - $44 471	> $44 471 - $53 492	> $53 492 – 136 191
Food insecurity rate	2.9% – 11.5%	> 11.5% - 14.4%	> 14.4% - 36.3%
Primary healthcare access rate (per 100 000 residents)	0 – 35.1	> 35.1 – 60.2	> 60.2 – 514.5
$PM_{2.5}$ concentration (μg/m^3)	4.04 – 7.38	> 7.38 – 8.49	> 8.49 – 14.98
Racial/ethnic minority rate	2.08% – 9.59%	> 9.59% - 27.82%	> 27.82% - 97.24%
Rurality rate	0 – 43%	> 43% - 75.3%	> 75.3% - 100%
High School Completion rate	26.4% – 85.2%	> 85.2% - 90.5%	> 90.5% - 98.9%

In this table we present the tertile values for each social and environmental determinant of health included in our study.

Abbreviations: SEDoH - social and environmental determinant of health

© 2024 Vieira de Oliveira Salerno PR et al. *JAMA Network Open.*

eTable 4. Relative Importance of Exposures

Exposure	R^2 (%) (95% confidence interval)
Median Household income	12.9 (1.7, 14.1)
Food insecurity rate	12.7 (10.8, 14.6)
High school completion rate	8.2 (6.6, 10.0)
$PM_{2.5}$	2.9 (2.1, 3.9)
Primary healthcare access rate	1.9 (1.3, 2.6)
Racial/Ethnic minority rate	1.4 (1.2, 1.8)
Rurality	0.9 (0.7, 1.2)

This table presents the ranked importance of the exposures as observed in our global model.

© 2024 Vieira de Oliveira Salerno PR et al. *JAMA Network Open.*

eFigure 8. Map of R^2 and *P*-Values From the Model

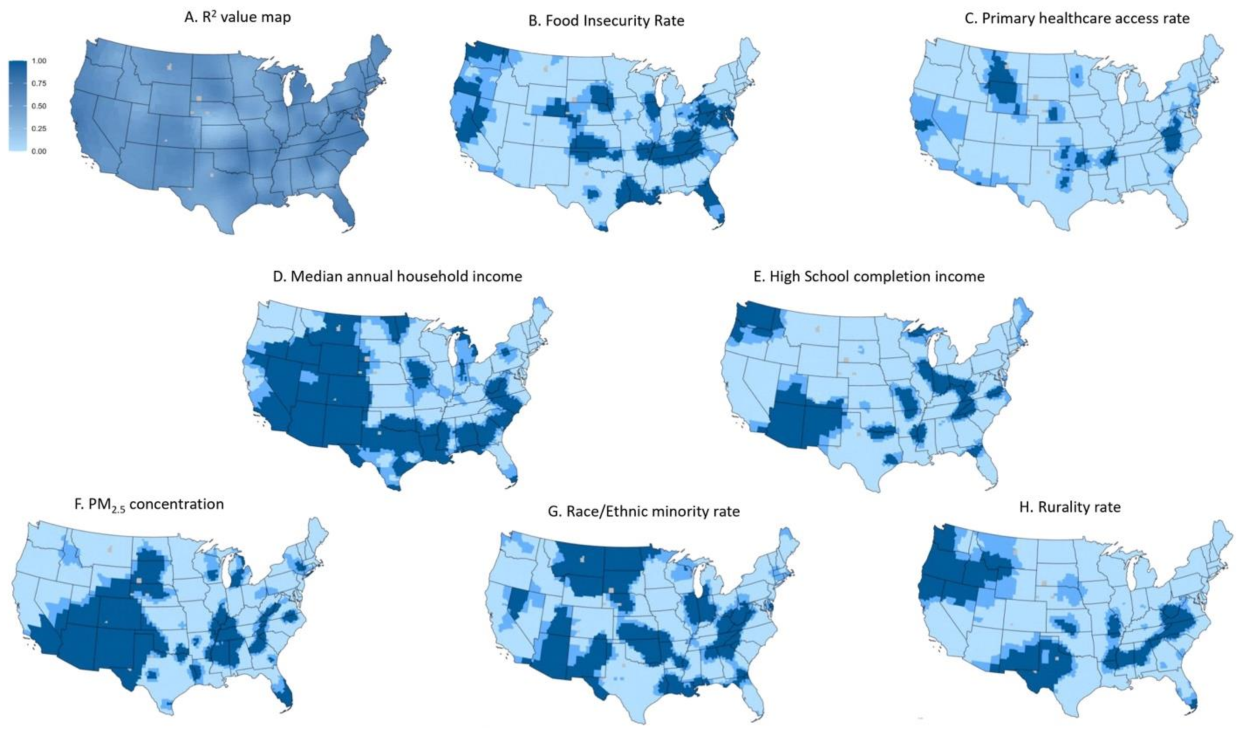

In these panel of maps, we present the results of the multivariable geographically weighted linear regression model fit to explore the association between county-level CKM related all-cause mortality rate and the studied SEDoH. (A) We present the model R^2 reported for each county in the US. A higher R^2 is better as that denotes more explanatory model fit. We observed that most counties reported an R^2 of 75% (0.75) or greater. The R^2 is reported on a continuous scale between 0 – 1 (0%-100%). A darker value represents a higher R^2 value for that county.

(B-H) We present the strength of association between the outcome and each exposure using p-values obtained for each county. Color code: light value $p > 0.05$ (not statistically significant), middle value $p < 0.05 – 0.01$ (statistically significant), and dark value $p < 0.01$ (highly statistically significant). These maps should be studied along with the map of model coefficients presented in Figure 3.

Abbreviations: CKM – cardio-kidney-metabolic syndrome

© 2024 Vieira de Oliveira Salerno PR et al. *JAMA Network Open.*
