# Supplementary material for: Social and Environmental Determinants of Health and Cardio-Kidney-Metabolic Syndrome–Related Mortality
Source: JAMA Netw Open. 2024 Sep 26;7(9):e2435783. doi: 10.1001/jamanetworkopen.2024.35783 (PMC11427959; doi:10.1001/jamanetworkopen.2024.35783)
Supplement: Supplement 2. — Data Sharing Statement [file jamanetwopen-e2435783-s002.pdf]

Data Sharing Statement

Vieira de Oliveira Salerno. Social and Environmental Determinants of Health and Cardio-Kidney-Metabolic Syndrome–Related Mortality. *JAMA Netw Open*. Published September 26, 2024. doi:10.1001/jamanetworkopen.2024.35783

Data
Data available: Yes
Data types: Data (not involving human participants)
How to access data: corresponding authors github page
When available: With publication

Supporting Documents
Document types: Statistical/analytic code
How to access documents: corresponding authors github page
When available: With publication

Additional Information
Who can access the data: all
Types of analyses: all analyses
Mechanisms of data availability: corresponding authors github page
